# *BMC Obesity* – expanding the *BMC* series into an important area of research

**DOI:** 10.1186/2052-9538-1-1

**Published:** 2014-02-19

**Authors:** Genevieve Horne, Philip McTernan, Tommy Visscher, Anna Peeters

**Affiliations:** BioMed Central, 236 Gray’s Inn Road, London, WC1X 8HB UK; Division of Metabolic and Vascular Health, Warwick Medical School, University Hospital Coventry and Warwickshire, Coventry, CV2 2DX UK; Research Centre for Obesity Prevention, Windesheim University of Applied Sciences, Zwolle, The Netherlands; Obesity and Population Health Department, Baker IDI Heart and Diabetes Institute, Melbourne, Australia

## Abstract

This Editorial marks the launch of an important new journal to join the *BMC* series portfolio – *BMC Obesity. BMC Obesity* joins *BMC Cancer* as the second journal within the series to focus on a particular condition in the human body and the factors that contribute towards it.

## Introduction

Being overweight or obese is now listed as one of the top five leading risks for death worldwide, with at least 2.8 million adults dying each year due to the associated risks of this condition. It is estimated that worldwide obesity has doubled since 1980, with pediatric obesity in itself becoming more and more of an issue; in 2011 for example, more than 40 million children under five were overweight [1].

The consequences of obesity are many and it is a serious risk factor for many chronic conditions such as heart disease, diabetes, and cancer, along with many other ailments such as sleep apnea, high blood pressure, and joint problems, not forgetting the grave impacts on psychological wellbeing. The problem of obesity was formally recognised as a global epidemic by a World Health Organization consultation in 1997 [2] and in recent years more and more funding has been made available for obesity related research to investigate how we can mitigate and prevent this global problem. It is estimated that 65% of the world’s population now live in countries where being overweight or obese is responsible for killing more people than being underweight or malnourished [1].

Obesity has many biological, social, economic, environmental, and behavioural factors contributing towards it – and the interactions between these are complex. Consequently the breadth of research being carried out into all of these is vast. Many organisations such as the National Institutes of Health (NIH), the Medical Research Council (MRC), the British Medical Association (BMA), and many others, both public, private, and federal, reflect this and make a wide range of funding available for the different branches of research, including molecular, genetic, behavioral, environmental, clinical, and epidemiologic studies [3].

With all this research being carried out and the scale of the obesity epidemic, we feel that there is a need for a new open access, open peer review, middle tier obesity journal for authors to publish their work in and discuss the new developments in this ever changing and fast moving field of research.

## Aims and scope

*BMC Obesity* considers articles on all aspects of human obesity across the lifecourse. These include the prevention, causes, treatment, management, and implications of obesity, incorporating lifestyle, epidemiology, policy and community/environmental interventions, related approaches for weight loss and the prevention of weight gain, management of the condition including the use of drugs and surgery, and basic science relating to the causes and effects of obesity. Research from developing country contexts is encouraged.

*BMC Obesity* will operate a system of open peer review as with the rest of the medical titles in the *BMC* series, as this is an important step in ensuring the transparency and fairness of the review process, and also allows reviewers to be given credit for their efforts. The open peer review process at BioMed Central means that authors can see who has reviewed their manuscript and once the article has been published, so can the journal’s readers, along with all the revisions and responses to the reviewer comments that are uploaded by the authors. All of the reports and author responses to these are published as part of the pre-publication history of the published article.

*BMC Obesity* has five editorial sections:

- □ Basic science, physiology, genetics, phenotyping and metabolism.
- □ Epidemiology and ethnicity.
- □ Lifestyle and community interventions.
- □ Policies, socioeconomic aspects, and health systems research.
- □ Treatment of obesity in clinical practice.

We are delighted to welcome Philip McTernan, Tommy Visscher, and Anna Peeters as Section Editors for the journal, along with a strong team of Associate Editors [4]. As the journal grows and develops, we will continue to recruit Editors to the board in order to adapt to the changing and growing nature of the field.

*BMC Obesity* aims to publish work deemed by peer reviewers to be a coherent and sound addition to scientific knowledge, and to provide an all important open access platform to allow the effective dissemination of this knowledge, so that we can all work together to more fully understand and address the obesity epidemic. Open access and the Creative Commons Attribution License [5] is vital in this, allowing universal and free access to all articles published in the journal and allowing them to be read and the data re-used without any restrictions.

*BMC Obesity* will work closely with the rest of the journals in the *BMC* series portfolio [6] to provide the best home for submitted manuscripts on obesity research. The ease of transferring manuscripts between the journals in the series helps to ensure that authors are able to reach the best audience for their paper and receive the maximum impact and readership that they can.

## The *Basic Science* section of *BMC Obesity*

We still have so much to understand about the field of obesity and so we are keen that in the *Basic science* section of *BMC Obesity* we don’t underestimate the versatility of the humble adipocyte, as it still has many secrets to tell us - as do many other cells. It would be nice to think that we have been successful in highlighting how important the adipocyte is to date, yet our grasp of how these cells, which can contain over 90% lipid, interact with other cells and tissues, is still proving complicated and challenging as the metabolism and secretome of the cells is so vast. However, technology and human ingenuity will no doubt lend a supportive hand to help delineate these interactions. The ability to build a more complex picture of human metabolism beyond the individual cell or organ will undoubtedly support the challenges to ensure we increasingly align research within a clinical perspective. These types of studies are challenging but we would be keen to see this area develop further, underpinned by murine studies as required.

Furthermore, it is apparent that whilst epidemiological studies have highlighted the impact of nutrition on our health, the detailed examinations of the effects of these nutrients have most often centred around vasculature, for obvious reasons. Whilst this has led to significant health benefits we would encourage further research looking beyond this to investigate the metabolic cellular health and the impact of nutrients on mechanisms such as mitochondrial dynamics, inflammation, and ER function as well as insulin and lipid signalling. These studies may highlight at an earlier phase how metabolic changes in various cell types may influence later metabolic dysfunction and disease risk.

We are keen that this journal becomes an effective concentrated resource for academics to come to rely on for high quality obesity science. It is also important that *BMC Obesity* provides quick and efficient access for researchers to source relevant publications, as they develop both their research concepts and papers for publication.

## The *Epidemiology and ethnicity* and *Policies, socioeconomic aspects, and health systems research* sections

As a complex condition with multiple causes and many adverse health outcomes, it is widely accepted that successful prevention of obesity will require a multi-stakeholder, multi-setting and multi-strategy approach. It will require novel approaches alongside extending and strengthening current approaches. It will require regular and reliable monitoring and evaluation and we are interested to see global examples of all these elements.

In the *Epidemiology and ethnicity* section we have a particular interest in novel uses of epidemiology to help us understand the evolution of the obesity trends, a focus on country to country and ethnic differences in these trends, and accurate estimation of the health burden also associated with these.

In the *Policies, socioeconomic aspects, and health systems research* section we are looking forward to both evaluations of implemented policies, and suggestions of novel policy approaches. We believe that limiting the impact of obesity on socio-economic inequalities in health will be critical, and so we would like to see articles that can shed new light on how this might be done. It is our hope that *BMC Obesity* will contribute to our ability to prevent the global increases in obesity through its role in making high quality, relevant research accessible to researchers, practitioners and policy makers around the world.

## The *Lifestyle and community interventions* section

During the last few decades highly sophisticated epidemiological, behavioural and interventional research has told us that the obesity epidemic is much more complicated than senior pioneer researchers had first thought and indeed had hoped [7]. New and innovative research is now suggesting that the ‘old methods’ are not sufficient to develop, implement and evaluate lifestyle and community interventions to efficiently combat the obesity epidemic. Whilst many of us, for example, were happy when interventionists realized that childhood prevention is successful only when parents are involved, we now claim to know that such interventions should be multidisciplinary, and even need integrated systems thinking [8]. Similarly, where many obesity researchers have celebrated reviewers’ comments when the reviewers have acknowledged that the randomized controlled trial is not the ultimate method to evaluate interventions, the literature is now rapidly moving forward past this and showing multifactorial, multilayer logic models [9], and multidimensional conceptual frameworks for integrated approaches [10].

Qualitative research is no longer deemed inferior to quantitative research. Perhaps most interesting is that innovative research methodologies are becoming very much practice-oriented. Research questions are being formulated together with professionals in the field, and the answers to these are much more focused towards really improving approaches to tackling obesity in the community. As a result, researchers are, and will be much more in the future, a lot closer to professionals in the field.

Still, although many of us are more and more involved in international meetings and international programmes on innovative approaches combating the obesity epidemic, there is little opportunity to publish new, innovative research opportunities. Reviewers are still insecure of appraising innovative methodologies that deviate from standard methods. The *Lifestyle and community interventions* section in *BMC Obesity* has the ambition to serve as an authority in the field for applied research in which professionals and researchers aim at developing new knowledge and experiences regarding obesity prevention, which can then be used in practice and aim at really combating the obesity epidemic. Whereas *BMC Public Health*, a related journal in the *BMC* series, has earlier proposed a research agenda with more applied or translational research [11], *BMC Obesity* welcomes and encourages such research with a focus on the world’s number one chronic disease threat for public health: obesity.
